# Permissive versus conventional weaning from venovenous ECMO in patients with ARDS: a retrospective cohort study

**DOI:** 10.1016/j.aicoj.2026.100174

**Published:** 2026-09-29

**Authors:** Quentin Moyon, Yoel Benitha, Chahem Harba, Maria Teresa Passarelli, Charles Juvin, Guillaume Lebreton, Juliette Chommeloux, Guillaume Hékimian, Alexandre Coppens, Ouriel Saura, David Levy, Charles-Edouard Luyt, Alain Combes, Matthieu Schmidt

**Affiliations:** a Department of Intensive Care, Institute of Cardiology, Pitié–Salpêtrière Hospital, Assistance Publique–Hôpitaux de Paris (AP–HP), Sorbonne University, Paris, France; b Department of Medicine and Surgery, Department of Emergency and Intensive Care, University of Milano-Bicocca, Fondazione IRCCS San Gerardo, Monza, Italy; c Department of Cardiac Surgery and Transplantation, Institute of Cardiology, Pitié–Salpêtrière Hospital, AP–HP, Sorbonne University, Paris, France; d Sorbonne University, INSERM, UMRS 1166–ICAN, Institute of Cardiometabolism and Nutrition, Paris, France; e Sorbonne University, GRC 30, RESPIRE, Paris, France

**Keywords:** Acute respiratory distress syndrome, Venovenous extracorporeal membrane oxygenation, ECMO decannulation, Weaning, Sweep gas-off trial

## Abstract

**Background:**

Criteria for weaning from venovenous extracorporeal membrane oxygenation (V-V ECMO) in acute respiratory distress syndrome (ARDS) remain poorly standardized. Some patients are decannulated despite persistent abnormalities in respiratory mechanics once oxygenation and carbon dioxide clearance are considered adequate. We aimed to compare outcomes associated with permissive versus conventional V-V ECMO weaning.

**Methods:**

We conducted a retrospective single-center cohort study including adult patients with refractory ARDS treated with V-V ECMO between January 2015 and December 2022 who were successfully decannulated while receiving invasive mechanical ventilation. Permissive weaning was defined as ECMO decannulation despite persistence of at least one of the following criteria during the sweep gas-off trial: plateau pressure ≥30 cmH₂O, driving pressure ≥20 cmH₂O, positive end-expiratory pressure ≥15 cmH₂O, or tidal volume ≤5 mL/kg predicted body weight. The primary outcome was 90-day mortality after ECMO decannulation. Secondary outcomes included liberation from mechanical ventilation within 90 days, analyzed using competing-risk models with death as a competing event. Associations with mortality were assessed using landmark Cox proportional hazards models anchored at decannulation.

**Results:**

Among 469 patients treated with V-V ECMO, 280 successfully decannulated patients were included; 117 (42%) underwent permissive weaning and 163 (58%) conventional weaning. Baseline characteristics were broadly similar, although body mass index was higher and SARS-CoV-2 ARDS more frequent in the permissive group. Patients undergoing permissive weaning had longer ECMO support (19 [9–37] vs. 12 [6–27] days; p < 0.01). At the sweep gas-off trial, respiratory mechanics remained more impaired in the permissive group, whereas oxygenation parameters were comparable. In landmark analysis, permissive weaning was associated with higher 90-day mortality in both univariable (hazard ratio [HR] 3.53, 95% confidence interval [CI] 1.62–7.66; p < 0.01) and multivariable analyses (adjusted HR 4.45, 95% CI 1.98–9.97; p < 0.01). However, permissive weaning was not associated with delayed liberation from mechanical ventilation by day 90 (adjusted subdistribution HR 0.90, 95% CI 0.70–1.17; p = 0.4).

**Conclusions:**

Among patients with severe ARDS successfully decannulated from V-V ECMO, permissive weaning was associated with increased 90-day mortality but not with delayed liberation from mechanical ventilation.

## Background

Veno-venous extracorporeal membrane oxygenation (V-V ECMO) is recommended for selected patients with refractory acute respiratory distress syndrome (ARDS) [1,2]. Randomized controlled trials, including CESAR [3] and EOLIA [4], as well as subsequent meta-analyses [5,6], have refined the indications for ECMO initiation. In contrast, criteria for V-V ECMO weaning remain poorly defined and lack standardization. Although expert consensus statements [7,8] and Extracorporeal Life Support Organization guidelines [9] provide general guidance, recent evidence from a scoping review and an international survey highlights substantial variability in weaning practices across centres [10,11].

Weaning from V-V ECMO in patients receiving controlled mechanical ventilation generally follows a stepwise approach. Readiness for weaning is typically considered when gas exchange can be maintained under protective ventilation settings, with a tidal volume of approximately 6 mL/kg predicted body weight (PBW), FiO₂ ≤60%, respiratory rate ≤28 breaths/min, PEEP ≤ 14 cmH₂O, and plateau pressure (Pplat) ≤28 cmH₂O, suggesting partial recovery of lung function [7]. A sweep gas-off trial (SGOT) is then performed, usually over several hours, during which oxygenation, carbon dioxide clearance, and ventilatory parameters are closely monitored. Decannulation is considered if predefined criteria are met, including PaO₂ ≥60 mmHg with FiO₂ ≤60%, PaCO₂ ≤50 mmHg, pH ≥7.36, RR ≤28 breaths/min, and Pplat ≤28 cmH₂O, in the absence doppler-echocardiography signs of right ventricular failure [4,7].

However, the decision to decannulate ultimately relies on clinical judgment. In practice, some patients are liberated from V-V ECMO despite not fulfilling all conventional criteria, either because further respiratory improvement is unlikely or due to ECMO-related complications. A single-centre study comparing conventional and permissive weaning strategies in 34 patients with COVID-19–associated ARDS reported similar mortality rates between groups, suggesting that current decannulation criteria may be overly restrictive in selected patients [12]. However, the small sample size and restriction to COVID-19 limit the generalizability of these findings.

The objective of this retrospective study was to compare 90-day mortality after ECMO decannulation between patients undergoing Permissive versus conventional V-V ECMO weaning in a large, unselected cohort. Secondary objectives included assessing the cumulative incidence of extubation after ECMO removal, with death treated as a competing event, and identifying factors independently associated with mortality.

## Methods

### Study design and patients

This study followed the STROBE (Strengthening the Reporting of Observational Studies in Epidemiology) recommendations for reporting cohort studies [13]. We conducted a retrospective cohort study at the Pitié-Salpêtrière Hospital, a tertiary referral ECMO centre in Paris, France. Adult patients with refractory ARDS who received V-V ECMO between January 2015 and December 2022 were screened for eligibility. Only patients successfully decannulated from V-V ECMO were included. For patients who underwent more than one ECMO run, only data from the first weaning episode were analysed. Exclusion criteria were use of ECMO as a bridge to lung transplantation; absence of invasive mechanical ventilation at the time of ECMO weaning; ECMO withdrawal as part of end-of-life decisions; and missing data regarding weaning strategy, date of death, or date of extubation. In accordance with the ethical standards of our Institutional Review Board, no informed consent was required for this retrospective, observational study. The National Commission for Informatics and Liberties (no. 2217028v0) approved the data collection for this study.

### ECMO management

V-V ECMO implantation and management followed local protocols consistent with international recommendations. Cannulation was performed percutaneously at the bedside by experienced operators using a femoral–jugular configuration as the first-line approach; when not feasible, a femoral-femoral configuration was used. For patients referred from other hospitals, a mobile ECMO retrieval team was deployed when appropriate. During ECMO support, mechanical ventilation was adjusted to minimize ventilator-induced lung injury, targeting ultra-protective lung ventilation settings, in line with protocols used in the EOLIA and CESAR trials [3,4]. Readiness for ECMO weaning was assessed by experienced ECMO physicians in accordance with international recommendations. The SGOT was protocolized and consisted of discontinuing sweep gas flow while maintaining ECMO blood flow at approximately 3 L/min. In patients who remained sedated and met potential weaning criteria, ventilation during the SGOT was typically delivered using a controlled mode with protective settings. The ability to breathe spontaneously was not required for ECMO weaning. The planned duration of the trial was 24 h.

### Weaning strategy classification

Conventional weaning was defined as ECMO decannulation following documented recovery of sufficient native lung function, evidenced by acceptable gas exchange and ventilatory parameters during a SGOT consistent with expert recommendations. Permissive weaning was defined as ECMO decannulation performed despite the persistence of at least one of the following criteria during the SGOT: plateau pressure ≥30 cmH₂O, driving pressure ≥20 cmH₂O, PEEP ≥ 15 cmH₂O, or tidal volume ≤5 mL/kg of PBW. This classification reflected a deliberate clinical decision to withdraw ECMO after adequate gas exchange had been achieved despite residual impairment in respiratory mechanics, including the need to maintain a reduced tidal volume to limit plateau and driving pressures, rather than waiting for full recovery of respiratory mechanics.

### Data collection

Data were collected using standardized case report forms and included: demographic characteristics; comorbidities; ARDS etiology; severity scores; pre-ECMO ventilatory and gas-exchange parameters; duration of mechanical ventilation before ECMO; ECMO configuration, duration, and complications; ventilatory parameters and arterial blood gases at the time of the SGOT; post-ECMO respiratory course; and ICU and hospital outcomes. Ventilator-free days to day 90 were calculated from ECMO decannulation, with zero ventilator-free days assigned to patients who died before day 90 or remained mechanically ventilated at day 90. When necessary, extubation dates were obtained from post-ICU rehabilitation facilities. Removal of a tracheostomy cannula was considered equivalent to extubation.

### Outcomes

The primary outcome was 90-day overall survival after ECMO decannulation. Secondary outcomes included the cumulative incidence of extubation within 90 days of decannulation, duration of post-ECMO mechanical ventilation, requirement for a new ECMO run, and ICU and hospital length of stay. All time-to-event analyses were anchored to the ECMO decannulation date.

### Statistical analysis

Categorical variables are reported as numbers (percentages) and compared using χ² or Fisher's exact tests, as appropriate. Continuous variables are reported as median [interquartile range] and compared using the Wilcoxon rank-sum test.

The primary analysis used Cox proportional hazards regression with a landmark at the time of ECMO decannulation to assess the association between weaning strategy and 90-day mortality. Follow-up was censored at 90 days post-decannulation. Kaplan–Meier curves were generated for visual comparison, and differences were assessed using the log-rank test.

The cumulative incidence of extubation was analysed using competing-risk methods, with death treated as a competing event. Cumulative incidence functions were estimated and compared between groups using Gray's test. Univariable and multivariable Fine–Gray subdistribution hazard models were used to assess the association between weaning strategy and time to extubation. Among patients alive at 90 days after ECMO decannulation, time to extubation was additionally described using Kaplan–Meier methods and compared between groups using the log-rank test.

For both the Cox and Fine–Gray models, multivariable analyses included the same prespecified covariates, selected a priori based on clinical relevance and the number of observed events: age, COVID-19 aetiology, use of renal replacement therapy during ECMO, and ECMO duration. Variables used to define the permissive weaning group, plateau pressure, driving pressure, PEEP, and tidal volume, as well as the duration of mechanical ventilation at the time of the SGOT, were not included in multivariable models to avoid collinearity, but were evaluated in univariable analyses. The proportional hazards assumption was verified using Schoenfeld residuals, and collinearity among covariates was assessed before model construction.

Missing data among covariates included in multivariable models were handled using complete-case analysis; the number of missing values for each variable is reported in Table S1. All analyses were performed using R (version 4.5.3). A two-sided p-value < 0.05 was considered statistically significant.

## Results

### Study population

Between January 2015 and December 2022, 469 patients with refractory ARDS were treated with V-V ECMO at our centre. Among the cohort, 288 patients were successfully weaned from ECMO. After excluding 8 patients, 2 due to missing data on weaning strategy classification and 6 who were extubated before ECMO decannulation, a total of 280 patients were included in the final analysis. Of these, 117 (42%) underwent permissive weaning and 163 (58%) conventional weaning, as detailed in Fig. 1. Most patients were weaned under controlled ventilation modes, with 200 (79%) receiving volume-controlled ventilation and 52 (19%) pressure-controlled ventilation. A total of 51 patients (18%) had two or more permissive weaning criteria, with “driving pressure ≥20 cmH₂O” and “Pplat ≥30 cmH₂O” being the most frequent, accounting for 67 (24%) and 61 (22%) patients, respectively (see Figure S1). The temporal distribution of permissive and conventional weaning strategies over the study period is shown in Figure S2.

**Fig. 1:**
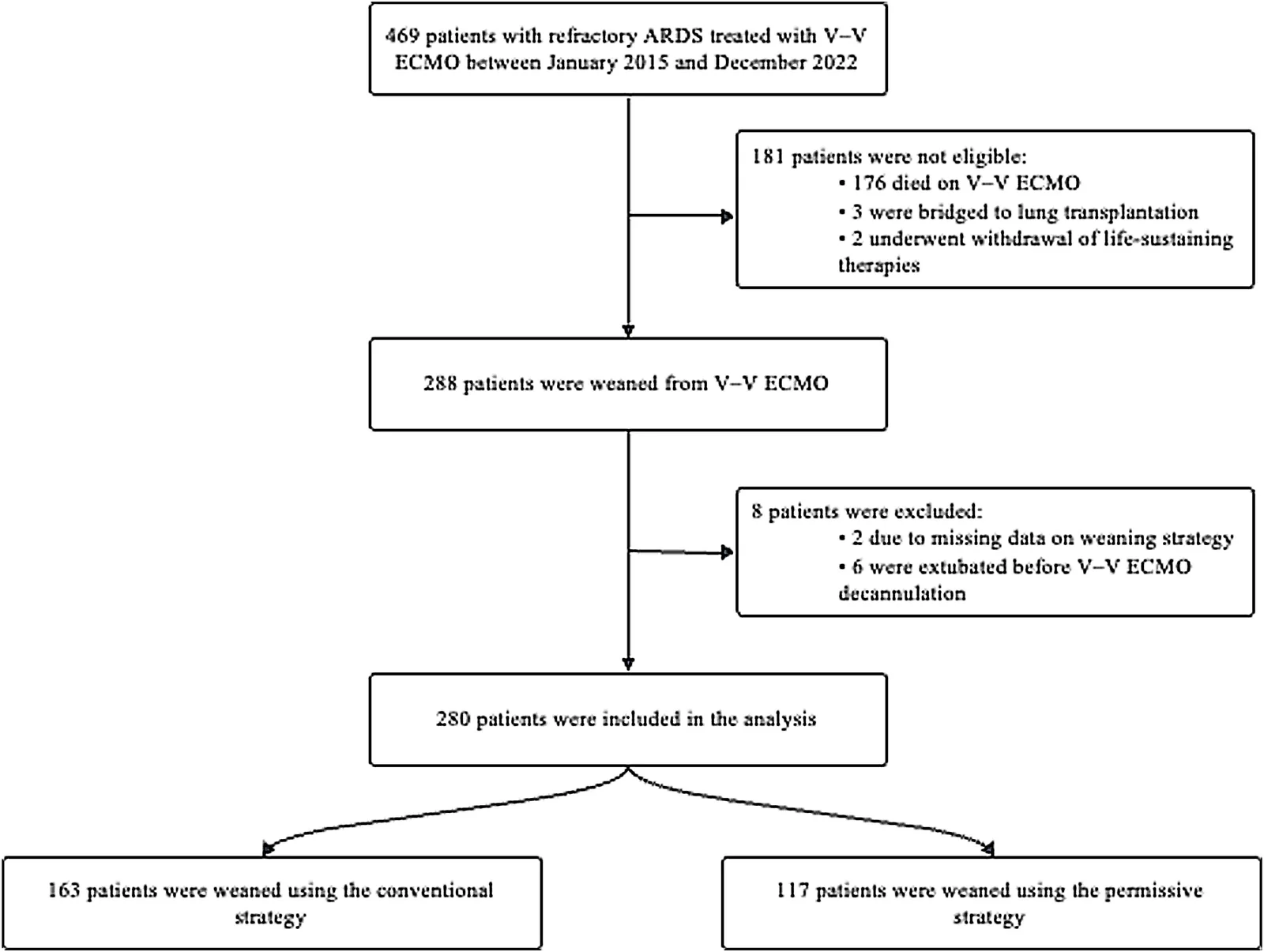
Study flow-chart.

### Characteristics at cannulation

Baseline characteristics at ECMO initiation were broadly similar between groups, as shown in Table 1. Median age was 46 [38–55] years in the permissive group and 50 [41–57] years in the conventional group, and approximately two-thirds of patients were male in both groups. Patients in the permissive group had a higher body mass index than those in the conventional group (35 [29–43] vs. 31 [28–35] kg/m²). ARDS was predominantly related to pneumonia in both groups, accounting for nearly 90% of cases overall, with a higher proportion of COVID-19–related ARDS in the permissive group (69% vs. 53%), as detailed in Figure S3. Markers of disease severity at ECMO initiation were comparable between groups. The SOFA score was similar, as were gas exchange parameters and ventilatory settings before ECMO initiation, including PaO₂/FiO₂ ratio, PaCO₂, respiratory rate, and respiratory system compliance. The use of adjunctive therapies before ECMO, including prone positioning, continuous neuromuscular blockade, nitric oxide, and renal replacement therapy, did not differ significantly between groups, as detailed in Table 1.

**Table 1:** Baseline characteristics of patients at ECMO cannulation according to weaning strategy.

Characteristic	Overall N = 280	Permissive weaning N = 117	Conventional weaning N = 163	p-value
Age, years	49 (39–56)	46 (38–55)	50 (41–57)	0.07
Male	186 (66)	78 (67)	108 (66)	0.9
Body Mass Index, kg/m²	32 (28–38)	35 (29–43)	31 (28–35)	<0.01
SOFA score before ECMO	12 (9–15)	12 (9–14)	12 (9–15)	0.6
Cause of ARDS				0.5
Pneumonia	249 (89)	106 (91)	143 (88)	
Unknown	19 (6.8)	8 (6.8)	11 (6.7)	
Autoimmune	8 (2.9)	3 (2.6)	5 (3.1)	
Other	4 (1.4)	0 (0)	4 (2.5)	
Aetiology of pneumonia				<0.01
SARS-CoV-2	148 (59)	75 (71)	73 (51)	
Bacterial	70 (28)	18 (17)	52 (36)	
Influenza	26 (10)	10 (9.4)	16 (11)	
Other pathogens	5 (2.0)	3 (2.8)	2 (1.4)	
MV duration before ECMO, days	3 (1–7)	4 (1–7)	3 (1–6)	0.07
PaO_2_/FiO_2_ ratio before ECMO	64 (55–75)	62 (55–70)	65 (54–78)	0.08
PaCO_2_ before ECMO, mmHg	57 (48–67)	59 (48–68)	55 (48–67)	0.4
Lactate before ECMO, mmol/L	1.6 (1.2–2.4)	1.6 (1.1–2.4)	1.6 (1.2–2.4)	1.0
PEEP before ECMO, cmH_2_O	12 (10–14)	12 (10–14)	12 (10–14)	0.3
Plateau pressure before ECMO, cmH_2_O	30 (29–32)	31 (30–32)	30 (29–32)	0.02
Driving pressure before ECMO, cmH_2_O	18 (16–20)	18 (16–20)	18 (15–20)	0.3
Tidal volume before ECMO, mL/kg PBW	6.0 (5.7–6.4)	6.0 (5.6–6.3)	6.0 (5.9–6.4)	0.3
Respiratory rate before ECMO, /min	30 (26–32)	30 (26–32)	30 (26–32)	1.0
Compliance before ECMO, mL/ cmH_2_O	21 (18–26)	21 (16–26)	22 (18–27)	0.1
Prone positioning before ECMO	230 (82)	100 (85)	130 (80)	0.2
Neuromuscular blockade before ECMO	274 (98)	113 (97)	161 (99)	0.2
Nitric oxide before ECMO	145 (52)	67 (57)	78 (48)	0.1
Corticosteroids before ECMO	36 (13)	15 (13)	21 (13)	>0.9
RRT before ECMO	34 (12)	15 (13)	19 (12)	0.8

ARDS – Acute Respiratory Distress Syndrome; ECMO – Extracorporeal Membrane Oxygenation; FiO₂ – Fraction of Inspired Oxygen; ICU – Intensive Care Unit; MV – Mechanical Ventilation; PaCO₂ – Partial Pressure of Carbon Dioxide; PaO₂ – Partial Pressure of Oxygen; PBW – Predicted Body Weight; PEEP – Positive End-Expiratory Pressure; RRT – Renal Replacement Therapy; SOFA – Sequential Organ Failure Assessment.

### ECMO course, weaning characteristics, and outcomes

Patients in the permissive group had a longer ECMO duration (19 [9–37] vs. 11 [6–23] days, p < 0.01) and a higher rate of ECMO-related complications, including the need of oxygenator exchange (42% vs. 27%, p = 0.01) and multiple ventilator-associated pneumonia episodes (≥2 episodes: 53% vs. 39%, p = 0.01) (Table 2). At the time of the SGOT, ventilatory parameters differed between groups, with a higher plateau pressure (29 [28–31] vs. 25 [23–27] cmH₂O, p < 0.01), higher driving pressure (20 [14–22] vs. 14 [[12], [13], [14], [15], [16]] cmH₂O, p < 0.01), and higher respiratory rate (28 [26–30] vs. 26 [24–30] breaths/min, p = 0.03) in the permissive group. In contrast, oxygenation was similar between groups, with comparable PaO₂/FiO₂ ratios (213 [174–278] vs. 196 [161–273] mmHg; p = 0.2) and no difference in FiO₂ distribution. Likewise, PaCO₂ did not differ significantly between permissive and conventional weaning at the end of the SGOT. The overall median tidal volume during the SGOT was 6.1 [5.8–6.4] mL/kg PBW, with observed values ranging from 3.8 to 9.8 mL/kg PBW. Mortality increased progressively, from 15.2% in patients with a single abnormal mechanical criterion to 66.7% in those with three (Figure S1). The association between individual respiratory parameters and 90-day mortality is presented in Figure S4.

**Table 2:** ECMO course, ventilatory parameters at the sweep gas-off trial, and clinical outcomes according to weaning strategy.

Characteristic	Overall N = 280	Permissive weaning N = 117	Conventional weaning N = 163	p-value*^2^*
ECMO course characteristics and complications				
Oxygenator exchange	91 (33)	48 (41)	43 (26)	<0.01
Any bleeding on ECMO	81 (29)	42 (36)	39 (24)	0.03
Ischemic stroke on ECMO	8 (3)	3 (3)	5 (3)	1.0
RRT during ECMO	107 (38)	49 (42)	58 (36)	0.3
Number of VAP episodes on ECMO				<0.01
0	72 (26)	17 (15)	55 (34)	
1	84 (30)	37 (32)	47 (29)	
≥2	124 (44)	63 (54)	61 (37)	
Cannulation infection on ECMO	58 (21)	29 (25)	29 (18)	0.1
Corticosteroids on ECMO	45 (16)	22 (19)	23 (14)	0.3
ECMO duration, days	14 (7–30)	19 (9–37)	11 (6–23)	<0.01
Respiratory parameters at the end of the sweep-gas off trial				
Ventilation mode				0.6
VCV	220 (79)	92 (79)	128 (79)	
PCV-APRV	52 (19)	23 (20)	29 (18)	
PSV	8 (3)	2 (2)	6 (4)	
FiO_2_				0.8
<40%	49 (18)	22 (19)	27 (17)	
40–60%	187 (67)	76 (65)	111 (69)	
≥60%	43 (15)	19 (16)	24 (15)	
Tidal volume, mL/kg PBW	6.1 (5.8–6.4)	6.0 (5.7–6.4)	6.1 (5.9–6.4)	0.07
RR, /min	28 (25–30)	28 (26–30)	26 (24–30)	0.02
PEEP, cmH_2_O	10 (8–14)	8 (8–15)	10 (8–12)	0.6
Plateau pressure, cmH_2_O	27 (24–29)	30 (28–31)	25 (23–27)	<0.01
Driving pressure, cmH_2_O	15 (13–19)	20 (15–22)	14 (12–16)	<0.01
Compliance, mL/ cmH_2_O	25 (20–33)	20 (16–26)	29 (33–35)	<0.01
PaO_2_/FiO_2_ ratio	201 (163–274)	210 (164–270)	197 (163–274)	0.6
PaCO_2_, mmHg	41 (38–45)	42 (38–46)	41 (37–45)	0.09
pH	7.43 (7.39–7.47)	7.43 (7.37–7.46)	7.43 (7.39–7.47)	0.05
Lactate, mmol/L	1.0 (0.7–1.3)	0.9 (0.7–1.3)	1.0 (0.7–1.4)	0.5
Outcomes				
New ECMO run after decannulation	14 (5)	6 (5)	8 (5)	0.9
Tracheostomy	120 (43)	61 (37)	59 (50)	0.030
Delay between tracheostomy and ECMO explant, days	4 (-10 – 19)	4 (-9 – 20)	2 (-10 – 16)	0.5
Prone positioning after ECMO	39 (14)	15 (9.2)	24 (21)	0.007
Corticosteroids after ECMO	19 (7)	12 (10)	7 (4)	0.05
Mechanical ventilation duration, days	43 (21–64)	47 (34–69)	36 (18–60)	<0.01
New intubation after ECMO decannulation	28 (10)	21 (13)	7 (6.0)	0.06
Number of VAP episodes after ECMO				0.5
0	107 (38)	41 (35)	66 (41)	
1	104 (37)	43 (37)	61 (38)	
≥2	67 (24)	32 (28)	35 (22)	
MV duration after ECMO decannulation, days	15 (7–30)	16 (9–30)	13 (6–29)	0.07
Ventilator-free days through day 90	74 (50–83)	67 (22–79)	76 (59–84)	<0.01
Hospital stays after ECMO explant, days	42 (25–74)	40 (24–76)	43 (28–73)	0.6
Hospital stays duration, days	73 (45–113)	74 (49–117)	72 (41–108)	0.3
Withdrawing life-sustaining treatment	26 (9)	14 (12)	12 (7)	0.2
Death at Day 90	31 (11)	22 (19)	9 (5)	<0.01
Cause of death				0.6
MOF	18 (56)	14 (61)	4 (44)	
Irreversible lung damage	5 (16)	3 (13)	2 (22)	
Other	9 (28)	6 (26)	3 (33)	

APRV – Airway Pressure Release Ventilation; ECMO – Extracorporeal Membrane Oxygenation; FiO₂ – Fraction of Inspired Oxygen; MOF – Multiple Organ Failure; MV – Mechanical Ventilation; PaCO₂ – Partial Pressure of Carbon Dioxide; PaO₂ – Partial Pressure of Oxygen; PBW – Predicted Body Weight; PCV – Pressure-Controlled Ventilation; PEEP – Positive End-Expiratory Pressure; PSV – Pressure Support Ventilation; RR – Respiratory Rate; RRT – Renal Replacement Therapy; VAP – Ventilator-Associated Pneumonia; VCV – Volume-Controlled Ventilation.

Day-90 mortality was higher in the permissive group (19% vs. 6%, p < 0.01), with multi-organ failure as the leading cause of death in both groups. Total duration of mechanical ventilation was longer in the permissive group (47 [34–69] vs. 36 [18–60] days, p < 0.01), whereas the duration of mechanical ventilation after ECMO decannulation did not differ significantly between groups (16 [9–30] vs. 13 [6–29] days, p = 0.07), as illustrated in Fig. 2. Ventilator-free days to day 90 were lower in the permissive-weaning group than in the conventional-weaning group (67 [22–79] vs 76 [59–84] days; p < 0.01). Hospital length of stay was similar between groups.

**Fig. 2:**
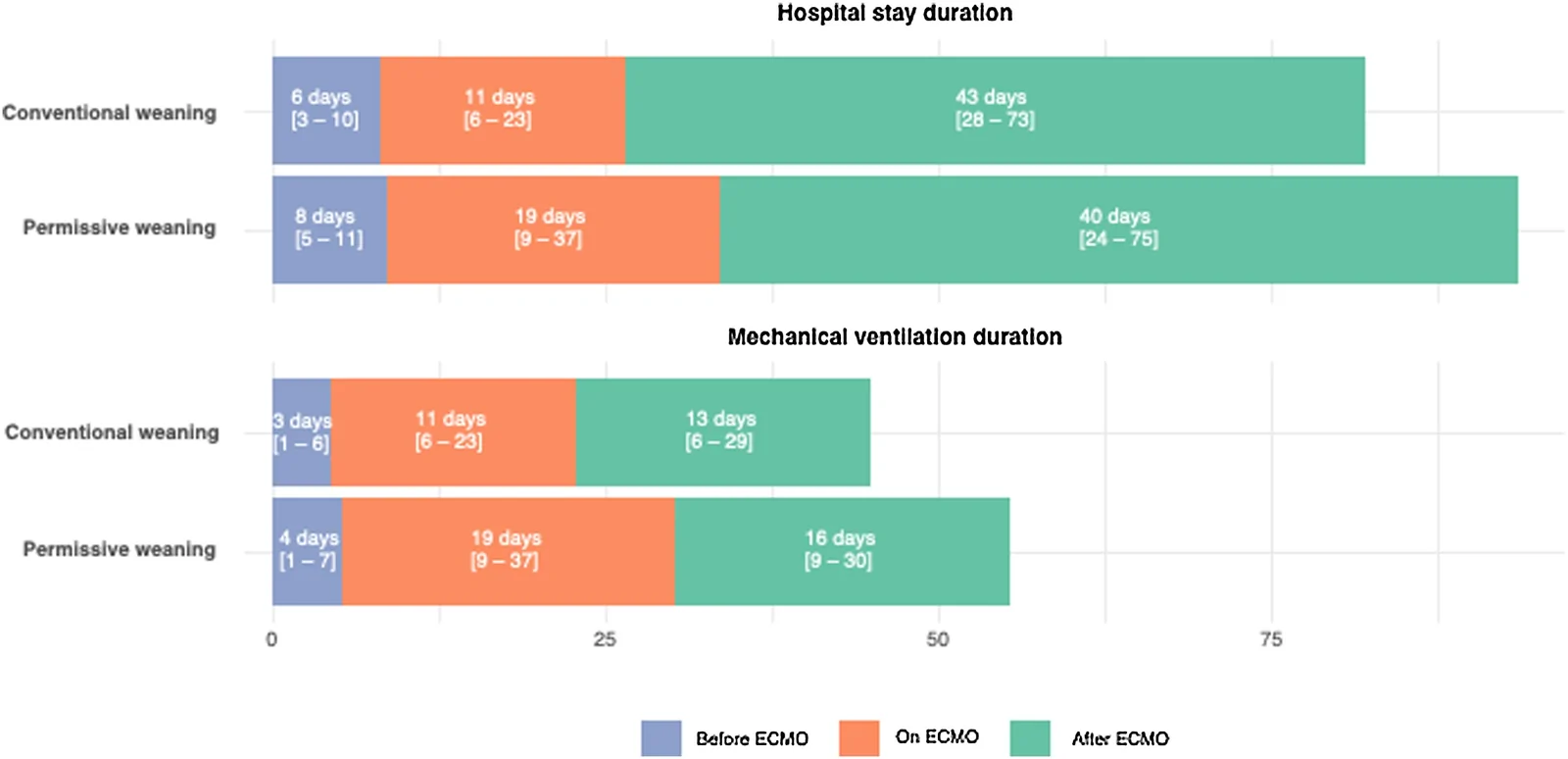
Median [interquartile] durations of hospitalization and invasive mechanical ventilation before, during, and after ECMO, stratified by weaning strategy.

### Day 90 mortality after ECMO decannulation

In the landmark Cox analysis anchored at ECMO decannulation, permissive weaning was associated with higher 90-day mortality. On univariable analysis, the hazard ratio for death in the permissive versus conventional group was 3.53 (95% CI 1.62–7.66, p < 0.01). After multivariable adjustment for age, COVID-19 aetiology, renal replacement therapy during ECMO, and ECMO duration, the association remained significant and increased in magnitude (HR 4.45, 95% CI 1.98–9.97, p < 0.01), as shown in Table 3 and Fig. 3. The proportional hazards assumption was met for all covariates. Results were consistent across sensitivity analyses in which each component of the permissive weaning definition was sequentially removed, with adjusted hazard ratios ranging from 2.56 to 5.26 (Table S2).

**Table 3:** Factors associated with 90-day mortality after ECMO decannulation: univariable and multivariable Cox proportional hazards analysis.

Characteristic	Univariable		Multivariable	
	HR (95% CI)	p-value	HR (95% CI)	p-value
Male	1.72 (0.74–3.99)	0.2		
Age, years	1.02 (0.98–1.05)	0.3	1.01 (0.98–1.04)	0.4
Body Mass Index, kg/m²	1.00 (0.96–1.04)	0.9		
Asthma or COPD	1.04 (0.45–2.41)	0.9		
ARDS subgroup				
COVID-19	–			
Bacterial	1.28 (0.50–3.25)	0.6		
Influenza	0.98 (0.22–4.38)	1.0		
Other	3.97 (1.71–9.18)	<0.01		
COVID-19	0.54 (0.26–1.11)	0.09	0.63 (0.29–1.39)	0.3
SOFA score before ECMO	1.20 (1.09–1.32)	<0.01		
RRT during ECMO	2.97 (1.42–6.20)	<0.01	2.26 (1.04–4.91)	0.04
Number of VAP episodes on ECMO				
0	–			
1	1.13 (0.48–2.65)	0.8		
≥2	0.51 (0.20–1.28)	0.2		
MV duration, days	0.97 (0.95–1.00)	0.02		
ECMO duration, days	0.98 (0.96–1.00)	0.10	0.98 (0.95–1.00)	0.06
Ventilation mode				
VCV	–			
PCV-APRV-BIPAP	1.86 (0.85–4.07)	0.1		
PSV	1.57 (0.21–11.69)	0.7		
Plateau pressure ≥30 cmH_2_O	2.65 (1.30–5.40)	<0.01		
PEEP ≥15 cmH_2_O	4.16 (1.96–8.84)	<0.01		
Driving pressure ≥20 cmH_2_O	1.51 (0.71–3.20)	0.3		
Tidal volume ≤5 mL/kg PBW	2.46 (0.75–8.09)	0.1		
Respiratory rate ≥30/min	1.15 (0.54–2.45)	0.7		
HCO3- ≥26 mmol/L	0.40 (0.19–0.81)	0.01		
Lactate ≥2 mmol/L	3.89 (1.36–11.11)	0.01		
FiO_2_ ≥60%	1.95 (0.87–4.37)	0.1		
PaO₂/FiO₂ ratio ≤ 150 mmHg	1.69 (0.76–3.78)	0.2		
Number of criteria for Permissive weaning				
0	–			
1	2.85 (1.16–7.02)	0.02		
2	3.88 (1.57–9.54)	<0.01		
3	12.96 (2.80–60.03)	<0.01		
4				
Permissive weaning status	3.53 (1.62–7.66)	<0.01	4.45 (1.98–9.97)	<0.01

ARDS, acute respiratory distress syndrome; BIPAP, bilevel positive airway pressure; CI, confidence interval; COPD, chronic obstructive pulmonary disease; ECMO, extracorporeal membrane oxygenation; FiO₂, fraction of inspired oxygen; HCO₃^−^, bicarbonate; HR, hazard ratio; MV, mechanical ventilation; PBW, predicted body weight; PCV-APRV, pressure-controlled ventilation–airway pressure release ventilation; PEEP, positive end-expiratory pressure; PSV, pressure support ventilation; RRT, renal replacement therapy; SOFA, Sequential Organ Failure Assessment; VAP, ventilator-associated pneumonia; VCV, volume-controlled ventilation.

**Fig. 3:**
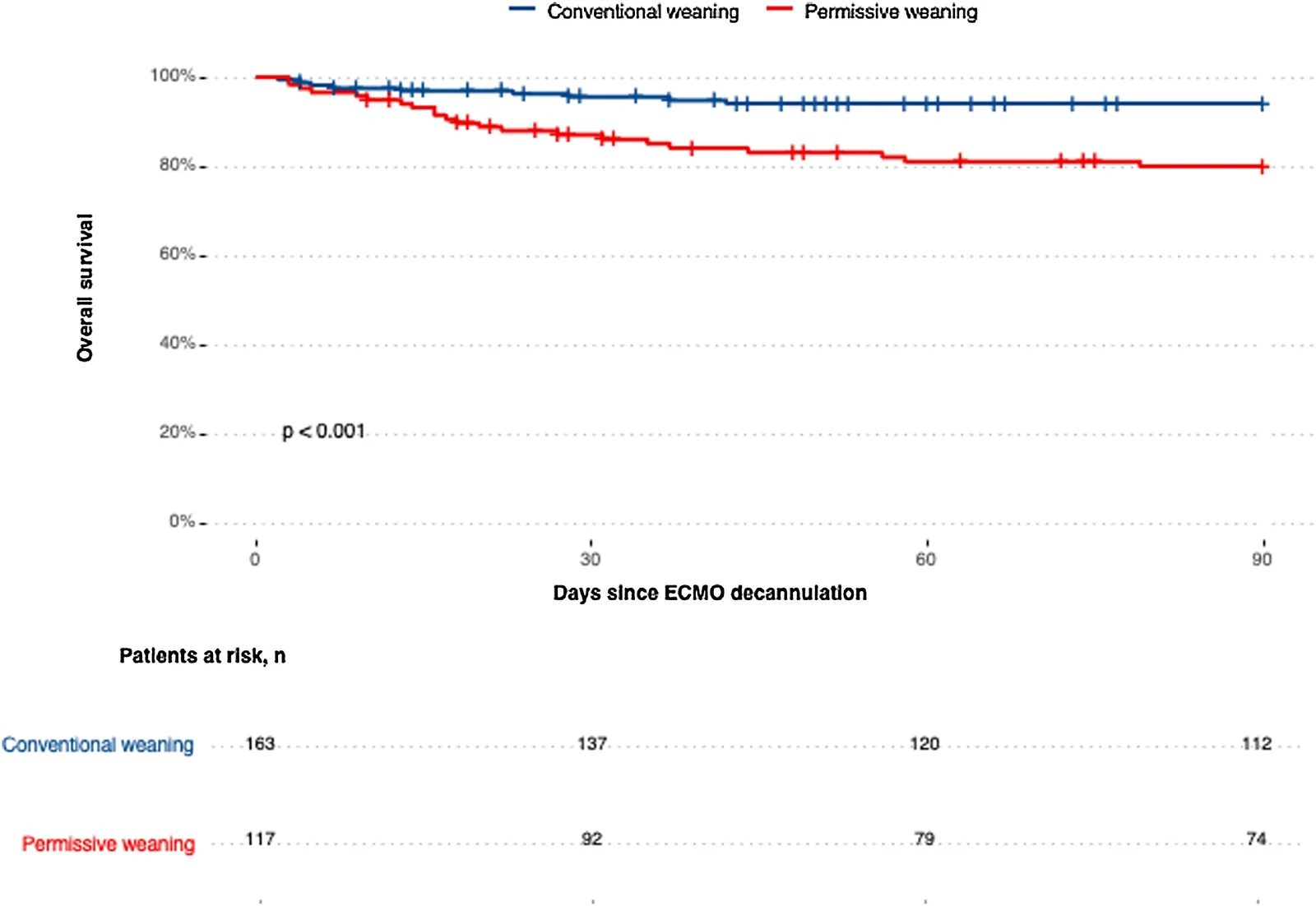
Kaplan Meier estimates 90-day survival after ECMO decannulation according to the weaning strategy.

### Time to extubation after ECMO decannulation

In the competing-risk analysis with death as a competing event, the cumulative incidence of extubation at 30, 60, and 90 days was 76.1%, 92.3%, and 95.7% in the Permissive group versus 77.3%, 93.9%, and 96.9% in the conventional group, respectively (Gray’s test p = 0.2), as shown in Figure S5.

Neither univariable (subdistribution hazard ratio (sHR) 0.85, 95% CI 0.67–1.07, p = 0.2) nor multivariable Fine–Gray analysis (sHR 0.90, 95% CI 0.70–1.17, p = 0.4), as detailed in Table S3, showed an association between weaning strategy and time to extubation. This finding was consistent when the analysis was restricted to 90-day survivors, among whom time to extubation did not differ significantly between groups (Figure S6). Notably, COVID-19-related ARDS was associated with a significantly lower probability of extubation by day 90 (adjusted sHR 0.76, 95% CI 0.59–0.98; p = 0.04), as were the need for renal replacement therapy during ECMO (adjusted sHR 0.69, 95% CI 0.53–0.88; p < 0.01) and higher age (adjusted sHR 0.98 per year, 95% CI 0.97–0.99; p < 0.01) (Table S3).

## Discussion

In this large cohort of patients with severe ARDS successfully decannulated from venovenous ECMO, permissive weaning despite persistent respiratory mechanical impairment was associated with increased 90-day mortality after decannulation, particularly when multiple abnormalities were present at the time of ECMO removal. In contrast, no significant difference was observed in the cumulative incidence of liberation from mechanical ventilation. Notably, despite a relatively stringent definition, permissive weaning was observed in 42% of patients, underscoring its frequency in routine clinical practice, even though oxygenation and carbon dioxide clearance were broadly similar between groups. Although the overall duration of mechanical ventilation was longer in the permissive weaning group, the duration of ventilation after ECMO decannulation did not differ from that observed with conventional weaning.

These findings expand on the small single-centre study by Masi and colleagues [12], who described the feasibility of ECMO decannulation despite persistent impairment in respiratory mechanics in patients with COVID-19-associated ARDS. As in their study, the permissive group in our cohort was characterized primarily by persistent abnormalities in respiratory mechanics rather than by markedly impaired oxygenation or CO_2_ clearance at the time of weaning. Taken together, these data suggest that, in selected patients, impaired respiratory mechanics alone may not preclude ECMO decannulation when gas exchange is considered acceptable, and the overall balance of risks favours withdrawal. Importantly, although permissive weaning was associated with higher mortality in our cohort, outcomes in this group remained within a range that does not justify systematically precluding ECMO decannulation solely based on persistent abnormalities in respiratory mechanics. Mortality increased progressively, from 15.2% in patients with a single abnormal mechanical criterion to 66.7% in those with three, suggesting that risk stratification may be better approached along a continuum rather than through binary thresholds. These findings support a more individualized strategy for ECMO discontinuation, integrating the burden of mechanical abnormalities with the presence of ECMO-related complications, rather than relying on rigid, mechanics-based weaning criteria.

Our results nevertheless differ from those previously reported [12] in two important respects. First, permissive weaning in our cohort was associated with a markedly higher post-decannulation mortality. Second, despite a longer total duration of mechanical ventilation, we did not observe a longer duration of mechanical ventilation after ECMO decannulation, nor a lower cumulative incidence of liberation from mechanical ventilation by day 90, including when analyses were restricted to 90-day survivors. Patients undergoing permissive weaning had fewer ventilator-free days to day 90. However, because patients who died before day 90 were assigned zero ventilator-free days, this difference was likely driven primarily by the higher mortality in this group rather than by slower liberation from mechanical ventilation, which was not supported by the competing-risk analysis. Several factors may explain these differences. The cohort by Masi et al. was restricted to COVID-19-associated ARDS and likely enriched for a fibroproliferative phenotype with persistently altered compliance and prolonged ventilatory dependence, whereas our cohort was larger, more heterogeneous, and not limited to SARS-CoV-2 infection. In addition, their definition of permissive weaning relied on a broader clinical judgment, including ECMO-related complications and prolonged support without improvement in respiratory mechanics, whereas our classification was based on prespecified ventilatory thresholds at the time of the SGOT trial.

An important finding of the present study is that the excess duration of total mechanical ventilation observed in the permissive group appeared to be mainly driven by a longer ECMO course rather than by prolonged ventilation after decannulation, as illustrated in Fig. 2. This point is supported both by the descriptive analysis and by the competing-risk analysis of extubation. In other words, patients in the permissive group spent more time invasively ventilated overall, but this was not clearly attributable to a slower ventilatory recovery after ECMO withdrawal. This distinction is relevant because it suggests that permissive weaning may identify a subgroup exposed to prolonged pre-decannulation support and higher overall severity, rather than a subgroup uniformly characterized by delayed liberation from mechanical ventilation once ECMO has been removed. The relatively long duration of ventilation after decannulation in both groups may also reflect determinants of extubation not captured in our database, including sedation, ICU-acquired weakness and impaired airway protection.

This study has several strengths. It includes a large cohort of patients decannulated from V-V ECMO, with detailed characterization of ventilatory parameters at the time of the SGOT and clinically relevant outcomes assessed up to 90 days. The use of time-to-event analyses, including competing risk models for liberation from mechanical ventilation, provides a more accurate assessment of post-decannulation trajectories. However, several limitations should be acknowledged. First, the retrospective design exposes the study to residual confounding and limits causal inference. Second, the definition of permissive weaning was based on predefined ventilatory thresholds derived from the available literature [3,4,7,8] and may not fully capture the clinical reasoning underlying ECMO withdrawal. Moreover, permissive weaning was retrospectively defined by the investigators and should therefore be interpreted as a marker of residual respiratory impairment at decannulation rather than as a formally applied clinical strategy. These residual abnormalities may reflect heterogeneous underlying processes, including unresolved lung injury and recurrent pulmonary infection, which this retrospective study was not designed to distinguish. Third, important physiological variables associated with weaning outcomes, such as ventilatory ratio, inspiratory effort, or PETCO_2_/PaCO_2_ ratio, were not available. Fourth, recannulation decisions may have been influenced by treatment-limitation orders, potentially underestimating early weaning failure. Fifth, most patients in our cohort were weaned under controlled ventilation, with only 3% receiving pressure-support ventilation at the time of decannulation, limiting the generalisability of our findings. Respiratory drive and inspiratory effort were not systematically measured and cannot be inferred reliably from the ventilation mode alone. An alternative strategy is to maintain ECMO until transition to pressure-support ventilation or spontaneous breathing, allowing these physiological variables to be assessed before decannulation. This was not standard practice in our centre, where ECMO withdrawal is prioritised once gas exchange is adequate to limit unnecessary exposure to ECMO-related complications. Moreover, available retrospective evidence has not demonstrated a clear benefit of delaying ECMO withdrawal until pressure-support ventilation is established [[14], [15], [16]]. Finally, the single-centre design in an experienced ECMO centre may further limit generalizability to other programs with less experience.

## Conclusion

In patients with severe ARDS successfully decannulated from V-V ECMO, discontinuation of extracorporeal support despite persistent abnormalities in respiratory mechanics was associated with increased 90-day mortality, particularly when multiple abnormalities were present at the time of decannulation. However, among survivors, this approach was not associated with delayed liberation from mechanical ventilation, indicating that meaningful pulmonary recovery may still occur despite residual mechanical impairment at the time of decannulation. These findings delineate a higher-risk subgroup in whom decisions regarding ECMO withdrawal should not rely solely on conventional respiratory mechanics criteria. Recognizing this risk profile may support a more individualized approach to decannulation and improve the accuracy of prognostic discussions, thereby helping clinicians provide clearer, more informed guidance to patients’ families and surrogate decision-makers.

## Authors’ contributions

QM, YB, and MS conceived and designed the study. YB, CH, and MP collected the data. QM, YB, and MS analysed the data and drafted the manuscript. QM and MS performed the statistical analyses. All authors critically revised the manuscript for important intellectual content and approved the final version.

## Consent for publication

Not applicable.

## Ethics approval and consent to participate

The study was approved by the French Data Protection Authority (Commission Nationale de l’Informatique et des Libertés, CNIL; authorization number 2217847v0). In accordance with French regulations for retrospective observational studies, the requirement for written informed consent was waived.

## Funding

No funding was received for this study.

## Availability of data and materials

The datasets used and/or analysed during the current study are not publicly available due to institutional and regulatory restrictions but are available from the corresponding author on reasonable request.

## Authors’ information

Not applicable.

## Declaration of competing interest

CEL reports lecture fees from Shionogi, grants from Eumedica and Merck, and travel support from Pfizer, all outside the submitted work. AC reports lecture fees from Getinge and Vantive. MS reports lecture fees from Getinge, Sedana, Solventum, Baxter, and Fresenius Medical Care outside the submitted work. The other authors declare that they have no competing interests.
