# Supplementary material for: Permissive versus conventional weaning from venovenous ECMO in patients with ARDS: a retrospective cohort study
Source: Ann Intensive Care. 2026 Sep 29;16:100174. doi: 10.1016/j.aicoj.2026.100174 (PMC13677267; doi:10.1016/j.aicoj.2026.100174)
Supplement: Supplementary file 1 [file mmc1.docx]

**Permissive versus conventional weaning from venovenous ECMO in patients with ARDS: a retrospective cohort study**

**AUTHORS:** Quentin Moyon^1^, MD*; Yoel Benitha^1^, MD*; Chahem Harba^1^, MD; Maria Teresa Passarelli^2^, MD; Charles Juvin^3^, MD, PhD; Guillaume Lebreton^3^, MD, PhD; Juliette Chommeloux^1^, MD; Guillaume Hékimian^1^, MD; Ouriel Saura^1^, MD; David Levy^1^, MD; Charles-Édouard Luyt^1,4^, MD, PhD; Alain Combes^1,4,5^, MD, PhD and Matthieu Schmidt^1,4,5^, MD, PhD

* Co-first authors

**INSTITUTIONS:**

1. Department of Intensive Care, Institute of Cardiology, Pitié–Salpêtrière Hospital, Assistance Publique–Hôpitaux de Paris (AP–HP), Sorbonne University, Paris, France
2. Department of Medicine and Surgery, Department of Emergency and Intensive Care, University of Milano-Bicocca, Fondazione IRCCS San Gerardo, Monza, Italy
3. Department of Cardiac Surgery and Transplantation, Institute of Cardiology, Pitié–Salpêtrière Hospital, AP–HP, Sorbonne University, Paris, France
4. Sorbonne University, INSERM, UMRS 1166–ICAN, Institute of Cardiometabolism and Nutrition, Paris, France
5. Sorbonne University, GRC 30, RESPIRE, Paris, France

Table of Contents

[TABLES	3](#_Toc232749909)

[Table S1. Missing data table	3](#_Toc232749910)

[Table S2. Sensitivity analyses of the association between permissive weaning and 90-day mortality after ECMO decannulation	4](#_Toc232749911)

[Table S3. Fine–Gray models for liberation from mechanical ventilation by day 90 from ECMO decannulation (death as a competing event)	5](#_Toc232749912)

[FIGURES	6](#_Toc232749913)

[Figure S1. Distribution of mechanical respiratory abnormalities criteria and association with 90-day mortality after V-V ECMO decannulation	6](#_Toc232749914)

[Figure S2. Temporal distribution of permissive and standard weaning among patients decannulated from V-V ECMO	7](#_Toc232749915)

[Figure S3. Causes of ARDS in study cohort	8](#_Toc232749916)

[Figure S4. Day-90 mortality according to respiratory parameters at the time of ECMO weaning	9](#_Toc232749917)

[Figure S5. Cumulative incidence of liberation from mechanical ventilation since ECMO decannulation	10](#_Toc232749918)

[Figure S6. Kaplan–Meier estimates of time to extubation among 90-day survivors after ECMO decannulation, according to weaning strategy.	11](#_Toc232749919)

# TABLES

## Table S1. Missing data table

| Variable | Missing (n) | Missing (%) |
| --- | --- | --- |
| Respiratory rate before ECMO, /min | 92 | 32.9 |
| Lactate before ECMO, mmol/L | 89 | 31.8 |
| Compliance before ECMO, mL/cmH2O | 75 | 26.8 |
| Tidal volume before ECMO, mL/kg | 69 | 24.6 |
| Driving pressure before ECMO, cmH2O | 68 | 24.3 |
| Plateau pressure before ECMO, cmH2O | 63 | 22.5 |
| PEEP before ECMO, cmH2O | 57 | 20.4 |
| PaCO2 before ECMO, mmHg | 52 | 18.6 |
| Hospital stay duration, days | 42 | 15.0 |
| Hospital stays after ECMO explant | 40 | 14.3 |
| Aetiology of pneumonia | 31 | 11.1 |
| PaO2/FiO2 ratio before ECMO | 11 | 3.9 |
| Cannulation infection on ECMO | 4 | 1.4 |
| Mechanical ventilation duration, days | 3 | 1.1 |
| SOFA score before ECMO | 2 | 0.7 |
| Oxygenator exchange | 1 | 0.4 |
| FiO2 at weaning | 1 | 0.4 |
| PaO2/FiO2 at weaning | 1 | 0.4 |
| pH at weaning | 1 | 0.4 |
| Lactate at weaning, mmol/L | 1 | 0.4 |

## Table S2. Sensitivity analyses of the association between permissive weaning and 90-day mortality after ECMO decannulation

| Definition of permissive weaning | N permissive (%) | Adjusted HR (95% CI) | p value |
| --- | --- | --- | --- |
| Primary definition (4 criteria) | 117 (42) | 4.45 (1.98–9.97) | <0.01 |
| Excluding PEEP ≥15 cmH₂O | 101 (36) | 4.09 (1.87–8.97) | <0.01 |
| Excluding tidal volume ≤5 mL/kg PBW | 111 (40) | 5.26 (2.34–11.83) | <0.01 |
| Excluding driving pressure ≥20 cmH₂O | 84 (30) | 2.56 (1.25–5.25) | 0.01 |
| Excluding plateau pressure ≥30 cmH₂O | 106 (38) | 4.37 (2.00–9.55) | <0.01 |
| CI, confidence interval; HR, hazard ratio; PBW, predicted body weight; PEEP, positive end-expiratory pressure. | | | |

## Table S3. Fine–Gray models for liberation from mechanical ventilation by day 90 from ECMO decannulation (death as a competing event)

| **Characteristic** | **Univariable (≤90 d)** | | **Multivariable (≤90 d)** | |
| --- | --- | --- | --- | --- |
|  | **sHR (95% CI)** | **p-value** | **sHR (95% CI)** | **p-value** |
| Male | 0.94 (0.74–1.20) | 0.6 |  |  |
| Age, years | 0.98 (0.97–0.99) | <0.01 | 0.98 (0.97–0.99) | <0.01 |
| Body Mass Index, kg/m² | 0.99 (0.98–1.00) | 0.2 |  |  |
| Asthma or COPD | 0.92 (0.69–1.22) | 0.5 |  |  |
| ARDS subgroup |  |  |  |  |
| *COVID-19* | – |  |  |  |
| *Bacterial* | 1.21 (0.89–1.64) | 0.2 |  |  |
| *Influenza* | 1.40 (0.98–2.01) | 0.06 |  |  |
| *Other* | 1.35 (0.89–2.06) | 0.2 |  |  |
| COVID-19 | 0.78 (0.61–0.99) | 0.04 | 0.76 (0.59–0.98) | 0.04 |
| SOFA score before ECMO | 0.98 (0.96–1.01) | 0.2 |  |  |
| RRT during ECMO | 0.73 (0.57–0.92) | <0.01 | 0.69 (0.53–0.88) | <0.01 |
| Number of VAP episodes on ECMO |  |  |  |  |
| *0* | – |  |  |  |
| *1* | 0.62 (0.43–0.91) | 0.01 |  |  |
| *≥ 2* | 0.48 (0.33–0.70) | <0.01 |  |  |
| MV duration, days | 0.99 (0.99–1.00) | <0.01 |  |  |
| ECMO duration, days | 0.99 (0.99–1.00) | 0.03 | 1.00 (0.99–1.00) | 0.08 |
| Ventilation mode |  |  |  |  |
| *VCV* | – |  |  |  |
| *PCV-APRV* | 0.78 (0.59–1.03) | 0.09 |  |  |
| *PSV* | 0.76 (0.33–1.74) | 0.5 |  |  |
| Plateau pressure ≥ 30 cmH2O | 0.80 (0.62–1.03) | 0.09 |  |  |
| PEEP ≥ 15 cmH2O | 0.89 (0.63–1.24) | 0.5 |  |  |
| Driving pressure ≥ 20 cmH2O | 0.91 (0.70–1.18) | 0.5 |  |  |
| Tidal volume ≤ 5 mL/kg PBW | 0.90 (0.40–2.00) | 0.8 |  |  |
| Respiratory rate ≥ 30/min | 0.68 (0.53–0.88) | <0.01 |  |  |
| HCO3- ≥ 26 mmol/L | 1.13 (0.89–1.43) | 0.3 |  |  |
| Lactate ≥ 2 mmol/L | 1.10 (0.50–2.42) | 0.8 |  |  |
| FiO2 ≥ 60% | 0.66 (0.48–0.93) | 0.02 |  |  |
| P/F ratio ≤ 150 mmHg | 0.83 (0.60–1.15) | 0.3 |  |  |
| Number of criteria for permissive weaning |  |  |  |  |
| *0* | – |  |  |  |
| *1* | 0.89 (0.67–1.19) | 0.5 |  |  |
| *2* | 0.82 (0.60–1.10) | 0.2 |  |  |
| *3* | 0.61 (0.25–1.50) | 0.3 |  |  |
| Permissive weaning status | 0.85 (0.67–1.07) | 0.2 | 0.90 (0.70–1.17) | 0.4 |
| ARDS, acute respiratory distress syndrome; CI, confidence interval; COPD, chronic obstructive pulmonary disease; COVID-19, coronavirus disease 2019; ECMO, extracorporeal membrane oxygenation; FiO₂, fraction of inspired oxygen; HCO₃⁻, bicarbonate; MV, mechanical ventilation; P/F ratio, arterial oxygen partial pressure to fraction of inspired oxygen ratio (PaO₂/FiO₂); PBW, predicted body weight; PCV-APRV, pressure-controlled ventilation–airway pressure release ventilation; PEEP, positive end-expiratory pressure; PSV, pressure support ventilation; RRT, renal replacement therapy; sHR, subdistribution hazard ratio; SOFA, Sequential Organ Failure Assessment; VAP, ventilator-associated pneumonia; VCV, volume-controlled ventilation. | | | | |

# FIGURES

## Figure S1. Distribution of mechanical respiratory abnormalities criteria and association with 90-day mortality after V-V ECMO decannulation

*Panel A: Overlap between predefined respiratory criteria at the time of ECMO weaning, including tidal volume ≤ 5 mL/kg predicted body weight (PBW), positive end-expiratory pressure (PEEP) ≥ 15 cmH₂O, driving pressure (DP) ≥ 20 cmH₂O, and plateau pressure (Pplat) ≥ 30 cmH₂O. Numbers within cells indicate the number of patients meeting each combination of criteria.*

*Panel B: Association between the number of abnormal mechanical criteria met at the time of ECMO decannulation and 90-day mortality. Bars represent mortality rates, with corresponding sample sizes indicated within each group.*

## Figure S2. Temporal distribution of permissive and conventional weaning among patients decannulated from V-V ECMO

*Bars represent the proportion of patients undergoing permissive or conventional weaning each year. Numbers within bars indicate the corresponding number of patients.*

## Figure S3. Causes of ARDS in study cohort

## Figure S4. Day-90 mortality according to respiratory parameters at the time of ECMO weaning

## Figure S5. Cumulative incidence of liberation from mechanical ventilation since ECMO decannulation

## Figure S6. Kaplan–Meier estimates of time to extubation among 90-day survivors after ECMO decannulation, according to weaning strategy.
